# Exploring the Spatial Determinants of Late HIV Diagnosis in Texas

**DOI:** 10.5888/pcd17.190346

**Published:** 2020-08-27

**Authors:** Sonia I. Arbona, Alassane S. Barro

**Affiliations:** 1 Texas Department of State Health Services, Austin, Texas; 2 Independent researcher, Austin, Texas

## Abstract

**Introduction:**

Despite statewide progress and continuous HIV prevention efforts in Texas, HIV diagnosis at a late stage of infection persists. Diagnosis delay differs in magnitude and spatial distribution. We examined the local spatial relationships of late HIV diagnosis with a selection of variables in an area of Texas that includes large metropolises and high HIV morbidity.

**Methods:**

We compared regression modeling approaches to study the associations between the regional percentage of late HIV diagnosis from 2011 through 2015, regional measures of poverty, lack of health insurance (uninsurance), educational attainment, unemployment, and the average regional distance from residence to an HIV testing site: global ordinary least squares linear regression, spatial error model, geographically weighted regression, and multiscale geographically weighted regression (MGWR). Cartographic representation of the local *R* ^2^, coefficient estimates, and their *t *values assisted in the interpretation of results.

**Results:**

The MGWR model resulted in a better fit and identified education and uninsurance as globally fixed predictors, whereas the relationships between late HIV diagnosis and poverty, unemployment, and distance varied spatially. The model performed better in rural areas and in suburban areas of the largest cities than in urban areas.

**Conclusion:**

The MGWR results provided local estimates of associations. The results highlight the importance of focusing on a local context. Modeling at the local scale is particularly useful for characterizing relationships between explanatory and dependent variables when the relationships vary spatially. In the context of HIV prevention, relationships that are of local relevance can inform local policy and complement routine screening in clinical settings.

**Summary**

**What is already known on this topic?**

HIV diagnosis at a late stage of infection persists in Texas. The rate of late HIV diagnosis varies by region but is generally lower in densely populated places with high HIV morbidity. These places have traditionally been the focus of HIV prevention.

**What is added by this report?**

Our study identifies regions where late HIV diagnosis occurs at a higher rate and explores associations with a selection of social determinants of health at those locations.

**What are the implications for public health practice?**

The results suggest distinct places for the development of local prevention strategies aimed at reducing late HIV diagnoses to further curb the HIV epidemic.

## Introduction

The introduction of highly active antiretroviral therapy in the 1990s to treat infection with the human immunodeficiency virus (HIV) transformed it into a treatable chronic infection for people with access to health care. HIV diagnosis followed by engagement in care has multiple benefits for individuals and their communities, including a better treatment outlook and a reduction in the probability of onward inadvertent transmission (1,2). Individual and community benefits prompted the Centers for Disease Control and Prevention (CDC) to recommend routine HIV testing in clinical settings for people aged 13 to 64 years, but the implementation of this recommendation has been gradual and slow. Despite continuous prevention efforts, and HIV testing becoming more widespread, thousands of people in the United States who are unaware of their HIV status do not present for HIV testing until late in infection (3,4).

Circumstances that affect HIV testing and diagnosis range from the individual to their communities and have been studied within the context of social determinants of health (SDOH) (5,6). These studies have made important contributions to our understanding of social and economic factors associated with HIV disease. Other studies have examined rural location (7–9) or have addressed the definition of late HIV diagnosis for cartographic representation (10). Although the rate of late HIV diagnosis varies by community, a spatial approach in the study of associated factors is relatively infrequent.

We focused on an area in the state of Texas characterized by dense population and high HIV morbidity. Our objective was to examine the local spatial relationships of SDOH factors, and a measure of distance, with late HIV diagnosis. The local spatial emphasis could provide a basis for prevention programs to develop local strategies that stress HIV testing initiatives tailored to the community.

## Methods

We compared regression modeling approaches to assess the global and local relationships between the percentage of late HIV diagnosis, socioeconomic indicators, and distance from residence to an HIV testing site. At a global scale, we used the global ordinary least squares (OLS) linear regression and further explored a global regression in a spatial error model. Simple linear regression assumes changes across a study area to be universal; variations across geographical space might be lost. At local scale, we used 2 modeling approaches: geographically weighted regression (GWR) and multiscale geographically weighted regression (MGWR). GWR allows parameter estimates to vary locally but does so with a single bandwidth. Potential issues with this approach are related to multiple hypothesis testing and accuracy. MGWR is an extension of the GWR method that allows multiscale modeling, eliminating the assumption that variations occur within the same scale. The approach is useful to gain information on the spatial scale of the associations. Model diagnostic statistics informed the selection of the regression model used in the analysis.

### Study area

Our study area included the 5 largest cities in Texas, by population and by HIV morbidity: Houston, Dallas, San Antonio, Austin, and Fort Worth. These metropolitan areas are funded by Part A of the Ryan White HIV/AIDS Treatment Extension Act of 2009 with a designation of Emerging Metropolitan Area (EMA) in Dallas and Houston and of Transitional Grant Area (TGA) in Austin, San Antonio, and Fort Worth (11). Our study area was defined by counties with an EMA or TGA designation and the counties that geographically lie between them. This created a continuous area where the network of interstate highways facilitates connectivity and interaction. In this area, distance from the largest urban centers to other populated places varied, as did the characterization of the population from predominantly urban to predominantly rural.

### Data regionalization

A regionalization method improved the precision of SDOH variables estimates originally obtained by census tract from the US Census Bureau American Community Survey (ACS) (12). ACS estimates for smaller geographical regions, such as census tracts, are based on a small sample of the population and result in large margins of error (13). To increase sample size by unit of analysis, our study followed the spatial approach of Spielman and Folch (14), which combined census tracts into larger regions through a spatial optimization algorithm. This approach sacrificed geographic detail but allowed us to specify a quality threshold to reduce the margins of error for each variable. This improved the precision of the regional estimates. The regionalization process created 345 regions within the study area. A region created from the regionalization of census tracts became the spatial unit of analysis.

### Data sources

Our study population consisted of people in the Texas Department of State Health Services Enhanced HIV/AIDS Reporting System who received a diagnosis of HIV disease during the 5-year period from 2011 through 2015, who were 18 years of age or older at the time of diagnosis, and who were not institutionalized. Late HIV diagnoses consisted of a subset of these cases (27%), defined as having received an AIDS diagnosis within 1 year of their first confirmed infection with HIV. We obtained the coordinates of the residential street address (91%) or zip code (9%) for all selected cases.

We sourced selected SDOH variables by census tract from the ACS 5-year estimates (2011–2015) (12). The definition of poverty was the proportion of the population aged 18 and older living below the US poverty level in the last 12 months of the survey response. The variable for educational attainment referred to the proportion of the population aged 18 or older with less than a high school diploma. Unemployment was defined as the proportion of the population aged 16 or older in the workforce without a job. Uninsurance referred to the proportion of the population aged 18 years and older without health insurance or a health coverage plan. A fifth variable measured the Euclidean distance from a person’s residence, or the residential zip code, to the closest HIV testing facility in the CDC National Prevention Information Network in 2017 (15). The average distance in each region resulting from the regionalization process defined the variable distance in the analysis, hereinafter referred to as distance.

### Spatial statistical models

**Global regression models**. An ordinary least squares (OLS) linear model explored the global associations between the independent variables and late HIV diagnosis. OLS models assume that observations are independent of one another, and the association between the predictor and response variables does not vary across the study area. This stationarity assumption implies that the relative contribution of each predictor variable (denoted as parameter β) to the variability in the response variable is the same in each geographical unit. OLS regression models also assume that residuals are identical and independently distributed. To check the assumptions of the OLS model, we used the following diagnostic tests: variance inflation factor to examine multicollinearity of explanatory variables, the Koenker (BP) statistic to assess the presence of local variation in the model, and the Moran’s I statistic to analyze the spatial pattern of the OLS regression residuals. We further examined global regression while controlling for spatial dependence in a spatial error model. Data manipulation and exploration used SAS version 9.4 (SAS Institute) and GeoDa version 1.12 (https://spatial.uchicago.edu/software). Global spatial model development and cartography used ArcGIS version 10.7.1 (ESRI).

**Geographically weighted regression (GWR) and multiscale GWR (MGWR) models.** Spatial processes may operate at local or global scales. We used the same set of variables included in the OLS model to specify GWR and MGWR models and to account for spatial nonstationarity in the relationships between the response (late HIV diagnosis) and predictor variables (SDOH characteristics and distance). GWR calibrates a regression model on each spatial unit of analysis and weights different regression parameters anywhere in the study area given a response and a set of predictor variables (16). GWR searches for an optimal bandwidth across all covariates and models all processes at the same spatial scale. MGWR extends the GWR framework by allowing different processes to operate at different spatial scales through optimal covariate-specific bandwidths (17). We used the MGWR V 2.1 software (https://sgsup.asu.edu/sparc/multiscale-gwr) for local spatial models.

Model specifications for both the GWR and MGWR calibrations were for a Gaussian linear model with an adaptive bi-square geographic kernel, and the local collinearity diagnostics. Estimates where values exceeded the critical threshold using a 95% confidence interval adjusted for multiple comparisons during parameter hypothesis testing. The adjustment was implemented as the ratio of the effective number of parameter estimates in the local model to the number of parameters in the global model (18,19).

## Results

**Figure 1:**
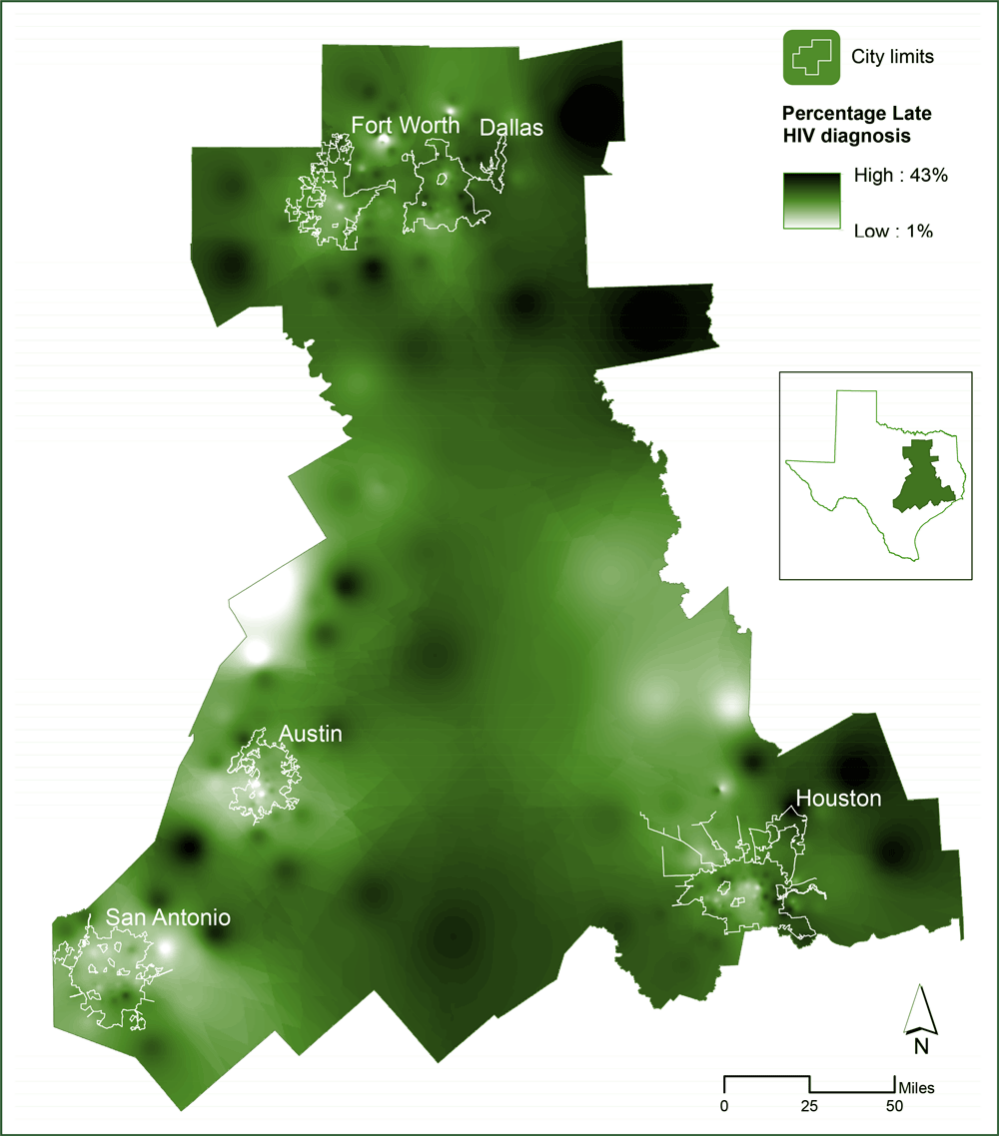
Spatially smoothed regional percentage of late HIV diagnoses in the study area in Texas (5 largest cities, by population and by HIV morbidity: Houston, Dallas, San Antonio, Austin, and Fort Worth).

The smoothed percentage of late HIV diagnoses varied spatially and ranged from 1% to 43% in the regions within the study area. Higher values concentrated in rural and suburban peripheral regions of the largest urban centers (Figure 1). The OLS regression model was significant (Wald statistic = 47.36, *P* < .001), and explained 14% of the variance in late HIV diagnosis in the study area. OLS results indicated that uninsurance (*P* = .005), poverty (*P* = .005), and distance (*P* < .001) were significantly associated with late HIV diagnosis whereas educational attainment (*P* = .37) and unemployment (*P* = .86) were not (Table 1). The highest variance inflation factor for the explanatory variables in the OLS regression model was 6.6, which, while not severe, signaled multicollinearity. The Koenker (BP) statistic was significant at a 95% confidence level (12.78, *P* = .03), indicating that the variance of the errors from the OLS model was nonstationary. The residuals of the OLS model were spatially correlated (Moran’s I = 0.11, *P* < .001), suggesting that the OLS model overestimated the percentage of late HIV diagnoses in some regions and underestimated it in others (Table 2). The spatial error model resulted in an improvement over the OLS model (AIC [Akaike Information Criterion] = 911, *R* ^2^ = 0.22), but it did not have a better fit than either the GWR or the MGWR models.

**Table 1:** Effects of Socioeconomic Variables and Distance on the Percentage of Late HIV Diagnoses at the Regional Level in Texas,^a^ 2011–2015

Variable	β Coefficient	Standard Error	*t* Value	*P* Value	VIF
Intercept^b^	0.24	0.009	28.21	<.001	^___^
Uninsurance	0.16	0.056	2.82	.005	6.6
Poverty	−0.13	0.046	−2.82	.005	4.1
Unemployment	0.02	0.110	0.18	.86	2.3
Educational attainment	0.04	0.043	0.89	.37	5.9
Distance	0.000002	0	4.03	<.001	1.1

Abbreviation: ___, not applicable; VIF, variance inflation factor.

a Study area included the 5 largest cities in Texas, by population and by HIV morbidity: Houston, Dallas, San Antonio, Austin, and Fort Worth.

b The intercept of the ordinary least square model defined as the expected value of percentage of late HIV diagnoses if all independent variables in the model are set to 0.

**Table 2:** Comparison of Regression Models on the Percentage of Late HIV Diagnoses at the Regional Level in Texas,^a^ 2011–2015

Comparison Statistic	Regression Model		
	OLS	GWR	MGWR
Adjusted *R* ^2^	0.14	0.51	0.51
AICc	917.51	815.24	789.23
Residual sum of squares	286.36	131.36	139.59
Moran's I of residuals	0.11, *P* < .001	0.01, *P* = .14	0.01, *P* = .18

Abbreviations: AICc, Akaike information criterion corrected; GWR, geographically weighted regression; MGWR, multiscale geographically weighted regression; OLS, ordinary least squares.

a Study area included the 5 largest cities in Texas, by population and by HIV morbidity: Houston, Dallas, San Antonio, Austin, and Fort Worth.

Both the GWR and MGWR models resulted in a higher adjusted *R* ^2^ value, explaining 51% of the overall variance of late HIV diagnosis, an important improvement from the OLS model. The GWR and MGWR models also had a significant drop in the residual sum of squares, and in the AIC corrected for small sample sizes (AICc). Of the 3 models, the MGWR had the lowest AICc (789.23), for an overall best fit (Table 2). Results in GWR had a slightly lower residual sum of squares than in MGWR. This, however, might be the result of model overfitting in GWR where the local intercept varies over space as opposed to an essentially constant intercept in MGWR. The GWR and MGWR models indirectly accounted for spatial autocorrelation resulting in a random spatial pattern of the residuals (GWR Moran’s I = .01, *P* = .14; MGWR Moran’s I = .01, *P* = .18). The local regional MGWR *R* ^2^ showed a range of explanatory power from 27% to 91% compared with the local regional GWR *R* ^2^ of 9% to 80%. The maximum possible bandwidth for the study area was 345. GWR yielded an optimal bandwidth of 66 while MGWR covariate-specific bandwidths varied (Table 3). The local condition number (local CN) provided a measure of local multicollinearity where the usual threshold value is in the range of 15 to 30. In the MGWR model all local conditions numbers were less than 10 while in the GWR model 11 regions had local condition numbers between 15 and 19 that, along with values for the local variance decomposition proportion and local variance inflation factor (local VIF), possibly indicated multicollinearity issues.

**Table 3:** Multiscale Geographically Weighted Regression Model Summary Statistics, Percentage of Late HIV Diagnoses at the Regional Level in Texas,^a^ 2011–2015

Diagnostic	Entire Model	Intercept	Uninsurance	Poverty	Unemployment	Education	Distance
Bandwidth	NA	44	336	133	44	336	88
Effective no. of parameters	52.98	18.19	1.55	3.83	16.86	1.65	10.87
Adjusted α^b^	.005	.002	.032	.013	.002	.030	.004
Adjusted *t* (95%)^c^	2.825	3.017	2.152	2.496	2.993	2.176	2.853

Abbreviation: NA, not applicable.

a Study area included the 5 largest cities in Texas, by population and by HIV morbidity: Houston, Dallas, San Antonio, Austin, and Fort Worth.

b Adjusted value of α.

c Adjusted critical *t *values using a 95% confidence interval.

**Figure 2:**
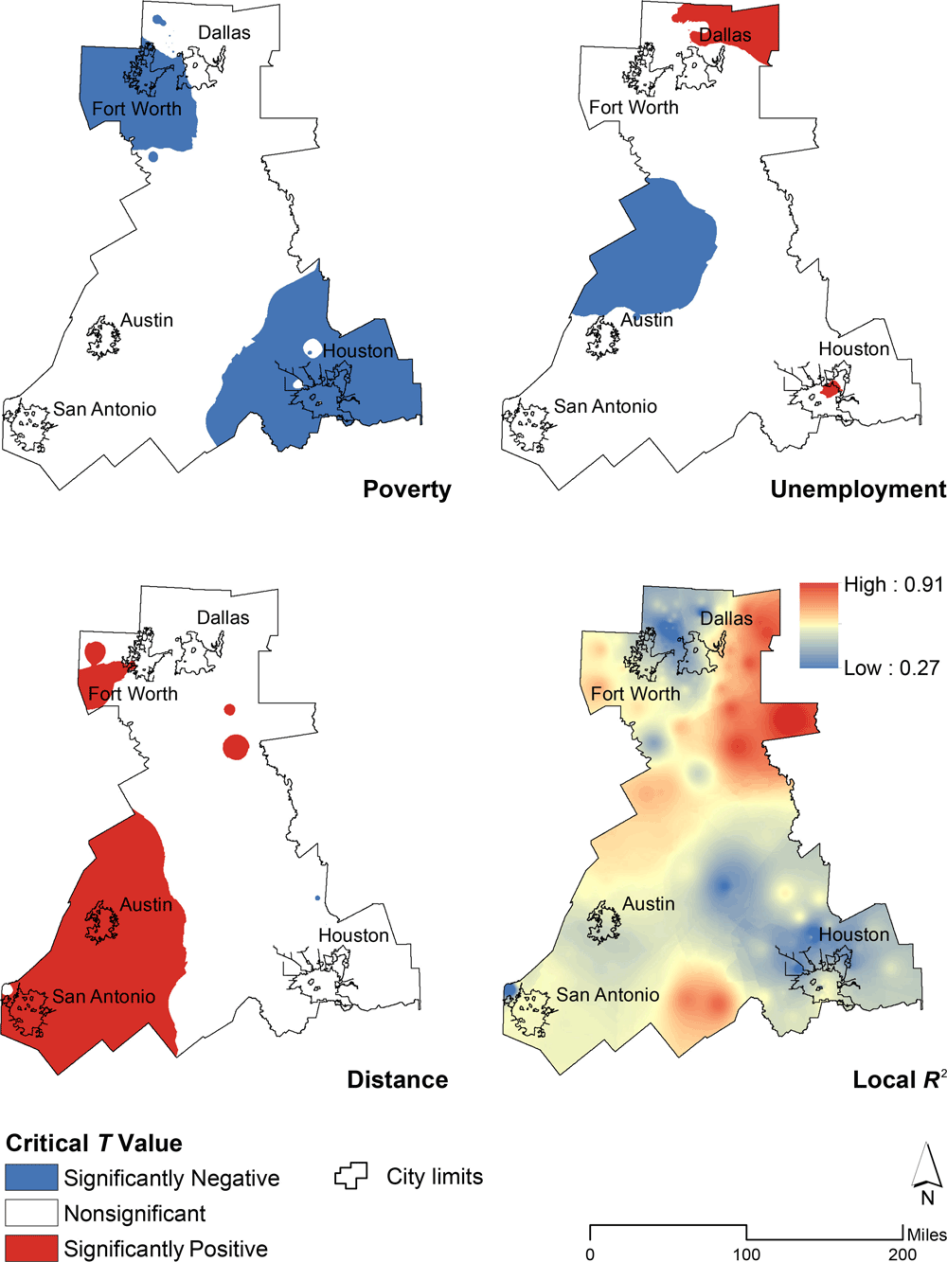
Spatial distribution of parameter estimates in study area (5 largest Texas cities, by population and by HIV morbidity: Houston, Dallas, San Antonio, Austin, and Fort Worth) of late HIV diagnosis at the regional level in Texas, 2011–2015. Maps show spatial distribution of parameter estimates for the percentage of people in poverty, percentage of people unemployed, distance to the nearest HIV testing site, and local *R* ^2^ in a multiscale geographically weighted regression.

After comparing the respective model results, we selected the MGWR model for the analysis. The model performed best in regions with relatively high rates of late HIV diagnosis (Figure 2). The effect of the parameters for uninsurance and educational attainment was positive and global in scale as indicated by their larger bandwidth of 336. These parameters were also positively associated in the OLS model, but only uninsurance was significant at the 95% confidence level. The parameters for poverty, unemployment, and distance had local effects. The cartographic representation of the local estimates shows regions where *t* values exceeded the 95% critical threshold.

In the global OLS model, the association of poverty with late HIV diagnosis was significantly negative; in the MGWR model, the effect and contribution from poverty was negative only in the northwest and southeast regions of the study area, including the cities of Fort Worth and Houston. These results suggest an association opposite to what is theoretically expected when low proportions of late HIV diagnosis are negatively associated with high levels of poverty.

Unemployment showed the most regional variation of all parameters as indicated by a bandwidth of 44. The local unemployment coefficients varied in the direction of their association with late HIV diagnosis; regions northeast of Dallas and within Houston had a positive association while regions at the center of the study area and north of Austin had a negative association.

The association between distance and late HIV diagnosis was positive in regions west of Fort Worth, southeast of Dallas, and in the southwest of the study area, including the cities of San Antonio and Austin. In a region north of Houston the association was negative. Regions positively associated fluctuated from predominantly rural to predominantly urban. The average distance to an HIV testing site was up to 26 miles (41,843 m) in some regions but less than 1 mile (1,609 m) in regions that include the large city centers.

## Discussion

Consistent with other studies in the United States, our study found that late HIV diagnosis was most prominent in rural areas (7,8). In our study, populated places in the periphery of large cities also had a comparatively high percentage of late HIV diagnoses. The results from our analysis contribute specific information about the extent of the spatial distribution of late HIV diagnosis, and the direction and strength of the associations with a set of explanatory variables.

Both the GWR and MGWR approach improved our understanding of the association between variables by highlighting locations in the association with parameters, but the MGWR model provided more insight on the scale at which parameters vary. The critical *t* value maps allowed visual inspection of specific associations with a strong influence in the model. The maps therefore suggest the varied importance of factors at the local level.

The concentration of HIV in economically disadvantaged urban populations has given rise to enhanced outreach efforts in poor urban communities at high risk of HIV transmission, potentially increasing HIV awareness and testing in these communities. The implementation of the CDC National HIV Behavioral Survey (NHBS) in the large metropolitan areas of Dallas and Houston may have further contributed to HIV awareness and testing in these regions. NHBS specifically targets urban areas with very high rates of poverty during its survey cycle of high-risk heterosexual residents. We presume that the combination of factors such as these has conditioned the effect of the variables under study, challenging the interpretation of complex multivariate associations.

It is in this context that we interpret the lower values in the percentage of late HIV diagnosis in regions that include the largest urban centers. Results from the MGWR align with this interpretation where negative association with poverty occurred in regions encompassing the metropolitan areas of Houston and Fort Worth. Other regions with high HIV morbidity, including Dallas, did not have a significant association with poverty.

The global effects of education and uninsurance are also particularly relevant in the interpretation of the association of unemployment with late HIV diagnosis. Regions where high unemployment was associated with a high percentage of late HIV diagnosis were in or near high population centers with the highest HIV morbidity. In these regions the absence of employer-based health insurance, the most common type of health insurance coverage, plausibly contributed to delayed HIV testing by lessening contact with a health care system. Publicly funded insurance may be an alternative to private or employer-based insurance, but unemployed people may not necessarily qualify for that type of insurance coverage. Regions at the center of the study area where unemployment had a negative relationship with late HIV diagnosis do not include the largest metropolitan areas. It is probable that in these regions the lack of employer-based health insurance, compounded by HIV unawareness and the underestimation of personal risk of HIV infection, hamper HIV testing, whether early or late.

HIV testing is generally free of charge or at low cost, thus diminishing financial barriers to the test. Beyond financial reasons, varied local conditions shape test-seeking and ease of access. In urban regions where residents are in close proximity to testing sites and morbidity is high, such as in San Antonio and Austin, these conditions probably go beyond the number of sites and their location to include community-specific factors such as choosing to travel longer distances to test, and perceptions of stigma (20). Although various factors contribute, access restricted by distance and available means of transportation affect the ability to reach HIV testing services, particularly for people who rely on publicly funded test sites. In those circumstances, increased distance presents a barrier to access the service (21,22). Longer travel time may result from difficulty in mobility. In urban regions in the study area, the extent of the urban public transport network varied, but most city dwellers rely on private cars for mobility. In rural regions, the barrier effect of travel time and cost on HIV testing may be further amplified by a low perception of risk (7). The local effect of distance may be negative or nonsignificant in regions with residents who regularly commute to cities with high HIV morbidity where they might be exposed to HIV outreach at the location of their daily activities rather than in their residential neighborhood.

The interpretation of our results is subject to limitations. Study findings are based on aggregate region-level data and should be considered exploratory. Our study did not consider demographic and HIV risk variables, which are known to affect the timing of HIV diagnosis. To explore the interaction of these variables would have required creating subsets of the HIV data into very small cell numbers. Also, although travel is not linear, the distance to HIV testing sites was measured as a straight line between place of residence and HIV testing site. Moreover, a single residential address cannot define all activity areas for a person.

Despite these limitations, our analysis contributes to better understanding of complex spatial variations of late HIV diagnosis in the context of an area with contrasting conditions, from large population centers with high HIV morbidity, high HIV awareness, and high levels of public health activities to other areas where morbidity, awareness, and public health activities are lower. Ideally, community assets and measures of hardship would also be considered in analyses such as ours because of how they can modify the exchange of information and resources, including those related to HIV prevention (22,23). The expense and time to collect information on local assets was not an option in our study, and it may not be practical everywhere. But where possible, it will enhance the understanding of local patterns and will benefit prevention efforts by helping to tailor HIV prevention activities to the locality.

To conclude, our study illustrates that a global approach to understanding the interactions between late HIV diagnosis, socioeconomic variables, and distance is insufficient because these interactions vary spatially. This information is of value to the allocation of limited HIV prevention resources, usually destined where they are most needed and where they can reach the most people. Ongoing efforts, such as the initiative, Ending the HIV Epidemic (EHE): A Plan for America, focus in their first phase on geographic hotspots of new HIV infections, including the large population cities in Texas that were part of our analysis. This is, and has been, a sensible public health approach that has benefitted communities most negatively affected by HIV. However, as initiatives like EHE recognize, the success of national public health strategies relies on disseminating efforts in HIV control beyond the hotspots, a dissemination better guided by a local-level focus. Thus, to further advance progress on the early diagnosis of HIV infection, it is imperative to continue the promotion of the key CDC recommendation to include voluntary HIV testing as a component of routine medical care regardless of individual risk factors and population HIV morbidity. Our analysis suggests that local suburban and rural communities are an appropriate focus for public health programs aimed at increasing HIV awareness and HIV testing among population groups most at risk for late HIV diagnosis. Our study suggests distinct places to further promote proven prevention strategies tailored to the local community.
